# Whole-Blood Transcriptome Analysis of Feedlot Cattle With and Without Bovine Respiratory Disease

**DOI:** 10.3389/fgene.2021.627623

**Published:** 2021-03-08

**Authors:** Janelle Jiminez, Edouard Timsit, Karin Orsel, Frank van der Meer, Le Luo Guan, Graham Plastow

**Affiliations:** ^1^Department of Agricultural, Food and Nutritional Science, Livestock Gentec, University of Alberta, Edmonton, AB, Canada; ^2^Department of Production Animal Health, Faculty of Veterinary Medicine, University of Calgary, Calgary, AB, Canada; ^3^Simpson Ranch Chair in Beef Cattle Health and Wellness, University of Calgary, Calgary, AB, Canada; ^4^Ceva Santé Animale, Libourne, France; ^5^Department of Ecosystem and Public Health, Faculty of Veterinary Medicine, University of Calgary, Calgary, AB, Canada

**Keywords:** bovine respiratory disease, differentially expressed genes (DEGs), host immune response, innate immunity, RNA sequencing

## Abstract

Bovine respiratory disease (BRD) is one of the main factors leading to morbidity and mortality in feedlot operations in North America. A complex of viral and bacterial pathogens can individually or collectively establish BRD in cattle, and to date, most disease characterization studies using transcriptomic techniques examine bronchoalveolar and transtracheal fluids, lymph node, and lung tissue as well as nasopharyngeal swabs, with limited studies investigating the whole-blood transcriptome. Here, we aimed to identify differentially expressed (DE) genes involved in the host immune response to BRD using whole blood and RNA sequencing. Samples were collected from heifers (average arrival weight = 215.0 ± 5.3 kg) with (*n* = 25) and without (*n* = 18) BRD at a commercial feedlot in Western Canada. RNAseq analysis showed a distinct whole-blood transcriptome profile between BRD and non-BRD heifers. Further examination of the DE genes revealed that those involved in the host inflammatory response and infectious disease pathways were enriched in the BRD animals, while gene networks associated with metabolism and cell growth and maintenance were downregulated. Overall, the transcriptome profile derived from whole blood provided evidence that a distinct antimicrobial peptide-driven host immune response was occurring in the animals with BRD. The blood transcriptome of the BRD animals shows similarities to the transcriptome profiles obtained from lung and bronchial lymph nodes in other studies. This suggests that the blood transcriptome is a potential diagnostic tool for the identification of biomarkers of BRD infection and can be measured in live animals and used to further understand infection and disease in cattle. It may also provide a useful tool to increase the understanding of the genes involved in establishing BRD in beef cattle and be used to investigate potential therapeutic applications.

## Introduction

Bovine respiratory disease (BRD) is one of the main causes of morbidity and mortality in beef cattle in North America (USDA, 2011). Beef cattle of all ages can be affected with BRD; however, they are most affected on or soon after entry into the feedlot (Babcock et al., 2010). This timing of infection is most likely due to the animal’s exposure to a wide range of pathogens that takes place at a time when various stressors (weaning, transportation, and commingling) negatively affect their immune system (Caswell, 2014; Timsit et al., 2016).

Although respiratory pathogens (mainly viruses and bacteria) and factors predisposing cattle to BRD are relatively well understood (Taylor et al., 2010), the host response and its relationship with disease outcomes to BRD, such as the host’s ability to maintain performance regardless of pathogen burden, needs to be further investigated (Van Eenennaam et al., 2014; Mulder and Rashidi, 2017). For instance, in cattle infected with respiratory pathogens, it is currently difficult to determine which cattle will exhibit visual and clinical signs of BRD or even require an antimicrobial treatment (Timsit et al., 2011b; Wolfger et al., 2015). Transcriptome analysis can lead to insights into disease processes, and biomarkers to assess disease states, progression, and prognosis. Thus far, transcriptomic techniques have examined bronchoalveolar fluids, lung tissue, and sputum samples of cattle with or without BRD (Aich et al., 2009; Rai et al., 2015; Behura et al., 2017; Johnston et al., 2019), but there is much less information on the whole-blood transcriptome (Lindholm-Perry et al., 2018; Sun et al., 2020). In comparison with lung tissue biopsies, blood is easier to obtain and can be collected repeatedly throughout the production period and can give real-time results, instead of postmortem conclusions. Furthermore the host immune response detected in the blood can reflect those responses occurring at the site of infection (Kawayama et al., 2016; Vinther et al., 2016).

Therefore, the objective of this study was to use RNA sequencing to analyze the whole-blood transcriptome of feedlot cattle with or without BRD. We hypothesized that animals exhibiting BRD would show a specific pattern of response in their blood transcriptome and that such patterns will provide further insight into the host immune response. Furthermore, variation in the blood transcriptome of animals with and without BRD could potentially provide markers of resistance or resilience markers for future application in breeding or management.

## Materials and Methods

### Ethics Statement

This study was conducted in accordance to the Canadian Council of Animal Care (2009) guidelines and recommendations (CCAC, 2009). All experimental procedures were reviewed and approved by the University of Calgary Veterinary Sciences Animal Care Committee (AC15-0109).

### Animals

Mixed-breed beef heifers at high risk of developing BRD (i.e., recently weaned, commingled, and auction-market derived) were enrolled between November 2015 and January 2016 at a commercial feedlot in Southern Alberta, Canada. At on-arrival processing, heifers received a subcutaneous injection of a long-acting macrolide (tulathromycin, Draxxin, 2.5 mg/kg, Zoetis, Kirkland, QC, Canada) and were weighed and vaccinated against infectious bovine herpes virus-1 (BoHV-1), bovine viral diarrhea virus (BVDV) (types I and II), bovine parainfluenza-3 (PI3V), bovine respiratory syncytial virus (BRSV), *Mannheimia haemolytica*, *Histophilus somni*, and clostridial pathogens. They were also dewormed with a pour-on ivermectin solution. In addition, they received a prostaglandin F2α analog to induce abortion, as per standard feedlot procedure. Heifers were fed in large outdoor dirt-floor pens with approximately 250–300 animals per pen. They were fed twice daily, a concentrate barley-based receiving/growing diet formulated to meet or exceed nutrient requirements. This diet contained 25 ppm of monensin (Rumensin 200, Elanco, Guelph, ON, Canada) and 35 ppm of chlortetracycline (Aureomycin 220, Zoetis). Each morning before feeding, bunks were visually inspected, and feed deliveries were adjusted to ensure that sufficient feed was available for *ad libitum* consumption. At approximately 30 days after arrival, cattle received another vaccination against infectious BoHV-1, BVDV types I and II, PI3V, BRSV, and a growth implant. Finally, cattle were individually weighed at approximately 120 days on feed (DOF). Average daily gain (ADG) was calculated using the difference between arrival weight and weight at blood sampling, divided by the DOF.

### Case Definition

Animals were retrospectively identified as BRD positive based on clinical examination and serum haptoglobin concentration. Heifers with at least one visual BRD sign, a rectal temperature ≥40°C, abnormal lung sounds detected at auscultation, a serum haptoglobin concentration ≥0.25 g/L, and no prior treatment against BRD or other diseases during the feeding period (i.e., first BRD occurrence) were defined as BRD cases. Heifers that had no visual signs of BRD, a rectal temperature <40°C, no abnormal lung sounds detected at auscultation, a serum haptoglobin concentration <0.25 g/L, and no history of treatment against BRD or other disease during the feeding period were treated as healthy controls, which were classified as non-BRD (NB) animals for transcriptome analysis.

### Study Design

Heifers were observed daily by experienced pen checkers for detection of clinical illness during the first 60 days from entry. Cattle with one or more visual signs of BRD (e.g., depression, nasal or ocular discharge, cough, tachypnea, or dyspnea) were removed from the pens by pen checkers and, if not previously treated for BRD or another disease during the feeding period, were clinically examined by an experienced veterinarian (ET) and a blood sample collected. For every heifer suspected of having BRD, one or two visually healthy cattle (no visual signs of BRD or other disease) were selected as pen-matched contemporary controls (for convenience, these animals were close to the gate or to the apparently sick animal, etc.) examined as for the BRD animals (if not previously treated for BRD or another disease during the feeding period).

Clinical examinations included assessment of visual signs of respiratory disease (cf. above), determination of respiratory rate and rectal temperature, and a complete lung auscultation using a conventional stethoscope to detect abnormal lung sounds (e.g., increased bronchial sounds, crackles, and wheezes). Two blood samples from each animal were collected at the same time by jugular vein puncture to determine (i) serum haptoglobin concentration [plastic serum tubes; Becton Dickinson, ON (Timsit et al., 2011a)] and (ii) the whole-blood transcriptome (Tempus tubes; Thermo Fisher Scientific, ON). Heifers with at least one visual BRD sign and a rectal temperature ≥40°C received an antibiotic treatment intramuscularly in combination with non-steroidal anti-inflammatory drugs (e.g., 40 mg/kg of florfenicol and 2.2 mg/kg of flunixin, 2 ml/15 kg, Resflor, Merck Animal Health) after sample collection, in accordance with feedlot treatment protocols.

### Determination of Serum Haptoglobin Concentration

Serum haptoglobin concentrations were determined in duplicate using a commercially available kit (Tridelta Phase Range Haptoglobin assay, Tridelta Development) as described (Timsit et al., 2011a). The working range was 0.0–2.5 g/L.

### Total RNA Isolation and mRNA Library Preparation

Total RNA was isolated from bovine blood using a Preserved Blood RNA Purification Kit (Norgen Biotek Corp, Thorold, ON, Canada), and the quality of RNA was measured using the 2200 RNA ScreenTape TapeStation System (Agilent Technologies Inc., Cedar Creek, TX, United States) producing RNA integrity (RIN) values ranging from 8.0 to 9.8. To prepare the mRNA cDNA libraries, 1.0 μg of total RNA was used from each sample using the TruSeq RNA Library Preparation kit v2 (Illumina, San Diego, CA, United States). Poly A-containing mRNA was enriched from the total RNA using poly-T oligo attached beads and fragmented for first-strand cDNA synthesis, followed by second-strand synthesis. The ends were repaired, and 3′ end adenylation and adapter ligation were performed for each library. Following these steps, libraries were polymerase chain reaction (PCR) amplified, validated using the Bioanalyzer (Agilent Technologies Inc., Cedar Creek, TX, United States), and finally normalized and pooled. Unique indices were used for all samples, and libraries were pooled and sequenced paired end (2 × 100 bp) on four separate lanes on a HiSeq 4000 platform, and sequencing was performed at McGill University and Genome Quebec Innovation Center (Montreal, QC, Canada). In total, 43 samples were used to generate paired-end sequences, and their raw reads were used for downstream analyses.

### Transcriptome Data Analysis

Raw reads were analyzed for quality and adapter sequence presence using FastQC (v0.11.8), and adapter sequences were removed using Trimmomatic (v0.39). These cleaned-up sequences were mapped and aligned to the *Bos taurus* reference genome (ARS-UCD1.2.98) using STAR (v2.7.1a) with default settings (Dobin et al., 2013), and read counts were generated using FeatureCounts (SubRead v1.6.4). The counts were then analyzed using the Bioconductor packages EdgeR and DESeq in the R (v3.5.2) software environment. Counts per million (CPM) was used to evaluate expression, and transcripts with CPM > 2 were considered as expressed.

### Differential Gene Expression Analysis

Differential gene expression results were obtained using EdgeR to compare animals with BRD (*n* = 25) with NB (*n* = 18) using the following parameters: *P*-value < 0.05 were adjusted to a 0.01 cutoff (*P*-adj), with a log fold change (Log2FC) > 2, with log CPM > 2. The data were also filtered with the “keep” command to keep samples with CPM ≥ 2 in at least 18 samples, as the number of samples in the NB group was 18 (Robinson et al., 2010). This value represents genes that are expressed in all the samples measured, and the dataset was normalized with the trimmed mean of *M*-values (TMM) normalization. To test for differential expression between the BRD and NB animals, the factors of “brd” and “pen” were used to test the difference in expression between the animals. The NB animals were set as the reference in this design model, and the read count data were fitted to a negative binomial general linear model (GLM) representing the design. Prior to fitting the model, the “Common,” “Trended,” and “Tagwise” negative binomial dispersion were estimated, and the biological coefficient of variation was calculated at 78% with a dispersion ratio of 0.61. Statistical tests were then performed for the coefficient relating to the BRD animals, and the top differentially expressed (DE) genes (DEGs) between the BRD and NB samples were ranked by *P*-value and absolute log2FC. In total, three different DEG analyses were performed: the total DEGs with read counts from both the BRD and NB animals (total DEGs, *n* = 43, coef = pen); BRD DEGs (*n* = 25, coef = cluster); and NB DEGs (*n* = 18, coef = pen). A “cluster” coefficient was also added for the BRD animals representing the three subgroups of the BRD samples differentiated by principal component analysis (PCA) determination of clustered samples (Cluster; *n* = 3).

### Ingenuity Pathway Analysis

Network and pathway analyses were analyzed using Ingenuity Pathway Analysis (IPA)^1^ (Qiagen, 2000–2019) software. This core analysis tool was used to identify gene pathways, disease, and networks using the gene expression data calculated by EdgeR. Input files of expression data included DEGs from all animals (*n* = 43) and the BRD-only animals (*n* = 25).

### Statistical Analyses

Statistical analysis used the R software package. *P*-values ≤ 0.05 were used to indicate significance, while false discovery rate (FDR) values were set at 0.05 for the adjusted *P*-values, unless otherwise stated. Both EdgeR and IPA incorporate statistical analyses into their analysis packages, and those values were reported. For ADG, rectal temperatures, and DOF, a Wilcoxon rank sum test with continuity correction was used to compare the BRD and NB animal values in the Dplyr package.

## Results

### Confirmation of Disease Status

Forty-four heifers (average arrival weight = 215.0 ± 5.3 kg) were enrolled to the study and were clinically examined and sampled between November 11 and December 11, 2015. Of these, 25 were classified as BRD positive and 18 were classified as NB based on clinical examination and serum haptoglobin concentration. One control heifer was removed from the study, as it had a serum haptoglobin concentration of 3.6 g/L (i.e., ≥0.25 g/L). Heifers with BRD had higher (*P* < 0.001) average rectal temperatures of 40.6 ± 0.03°C, than NB heifers averaging 39.3 ± 0.14°C (Supplementary Table 1). Furthermore, the ADG in the NB heifers was considerably higher (*P* < 0.001) than in the BRD heifers, which on average gained less weight (*P* < 0.001) from the time they arrived to the feedlot to the time they were enrolled in the study (Supplementary Table 1).

### Total Gene Expression Data Summary of All Bovine Respiratory Disease Animals Compared With All Non-bovine Respiratory Disease Animals

**FIGURE 1:**
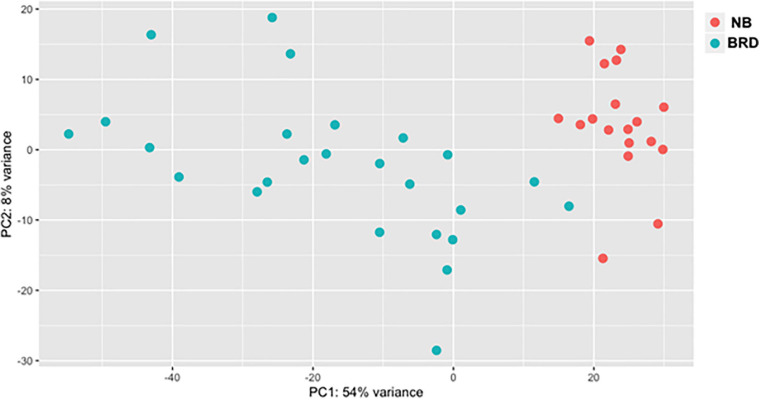
Principal component analysis (PCA) plot comparing differences in total differentially expressed (DE) gene populations between bovine respiratory disease (BRD) and non-BRD (NB) animals. PCA plot displaying differing clustering patterns between heifers displaying clinical signs of BRD (blue) and non-BRD animals (red). Plot was designed using normalized counts (*n* = 43), using the variable stabilization transformation for the PlotPCA tool in DEGSeq.

A total of 1.51 billion raw reads were generated for the mRNA libraries, and after trimming, an average of 31 M reads per sample was used for alignment (Supplementary Table 2). The read-mapping rates ranged from 75.27 to 92.09%, and on average approximately 25 M reads were uniquely mapped per sample (Supplementary Table 3). In total, EdgeR analysis identified 11,966 genes, with 3,075 downregulated and 3,236 upregulated when comparing the BRD with NB samples (*n* = 43) using BRD as the coefficient to determine DEGs; 6,311 total DEG, log2FC > 2, *P*-adj < 0.05. To explore the difference between the expression profiles of the NB and BRD samples, PCA was used to analyze the differences and similarities between the samples. The PCA showed that whole-blood transcriptome profiles of BRD cattle were separated from the NB profiles with 54% of the variation attributed to PC1 (Figure 1). Four samples appear as outliers in the PCA plot: two BRD samples and two NB samples (Figure 1). As might be expected from this result, the number of DEGs in the NB group was relatively small (*n* = 33 DEGs; total transcripts = 11, 787), whereas thousands of DEGs were identified within the BRD samples, which had a total of 13,404 transcripts identified.

### Identification of the Differentially Expressed Genes Between Bovine Respiratory Disease and Non-bovine Respiratory Disease Animals

To investigate the host response due to BRD infection, the top ranked DEGs were identified by comparing the DEGs between the NB and BRD samples. Table 1 shows the genes with the highest logFC values using the NB animal expression as the reference. Major immune genes such as *interleukin* (*IL*)*1 receptor 2* (*IL1R2*), *complement factor B* (*CFB*), and *IL3 receptor subunit alpha* (*IL3RA*) were identified in the top 10 upregulated DEGs, with *TNF alpha induced protein 6* (*TNFAIP6*) and *IL12B* evident in the top 30 upregulated DEGs. Furthermore, *haptoglobin* (*HP*), *lipocalin* (*LCN2*), *serpin peptidase inhibitor* (*SERPINB4*), and *S100 calcium-binding proteins* (*S100A9* and *S100A8*) were also among the top expressed genes in the BRD animals (Table 1). The top downregulated DEGs when comparing the BRD with NB animals (Table 2) belonged to hemoglobin synthesis pathways, including *alpha globin* (*HBA*), *beta globin* (*HBB*), *mu globin* (*HBM*), and *aminolevulinic acid synthase* (*ALAS2*). The enriched genes (upregulated in the BRD animals) belong to immune response pathways, as well as gastrointestinal, inflammatory, infectious, and respiratory disease pathways (not shown).

**TABLE 1:** Top enriched total differentially expressed (DE) genes identified when comparing all bovine respiratory disease (BRD) with all non-BRD (NB) animals.

**Gene name**	**Gene description**	**LogFC**	***P*-Adjust**
*LRG1*	Leucine-rich alpha-2-glycoprotein 1 [Source: VGNC Symbol; Acc: VGNC:30980]	7.84	5.55E-29
*SERPINB4*	*Bos taurus* serpin peptidase inhibitor, clade B like (LOC786410), mRNA. [Source: RefSeq mRNA; Acc:NM_001206713]	6.14	1.18E-19
*IL1R2*	Interleukin 1 receptor type 2 [Source: VGNC Symbol; Acc: VGNC:30132]	5.82	2.71E-20
*EREG*	Epiregulin [Source: VGNC Symbol; Acc: VGNC:28575]	5.37	3.14E-22
*THY1*	thy-1 cell surface antigen [Source: VGNC Symbol; Acc: VGNC:35856]	5.26	8.05E-20
*CFB*	Complement factor B [Source: NCBI gene; Acc: 514076]	4.74	1.05E-28
*DCSTAMP*	Dendrocyte expressed seven transmembrane protein [Source: VGNC Symbol; Acc: VGNC:27925]	4.25	1.80E-22
*BMX*	BMX non-receptor tyrosine kinase [Source: VGNC Symbol; Acc: VGNC:26529]	4.14	2.65E-30
*DPYS*	Dihydropyrimidinase [Source: VGNC Symbol; Acc: VGNC:28194]	4.11	9.58E-19
*IL3RA*	Interleukin 3 receptor subunit alpha [Source: NCBI gene; Acc: 100299249]	4.10	1.17E-31
*ADGRG3*	Adhesion G protein-coupled receptor G3 [Source: VGNC Symbol; Acc: VGNC:25667]	4.01	8.40E-37
*TNFAIP6*	TNF alpha induced protein 6 [Source: VGNC Symbol; Acc: VGNC:36156]	3.64	7.16E-17
*MMP9*	Matrix metallopeptidase 9 [Source: VGNC Symbol; Acc: VGNC:31531]	3.58	1.94E-14
*CLEC1B*	C-type lectin domain family 1 member B [Source: VGNC Symbol; Acc: VGNC:58366]	3.50	3.98E-13
*PLA2G4F*	Phospholipase A2 group IVF [Source: VGNC Symbol; Acc: VGNC:32962]	3.47	3.30E-25
*LCN2*	Lipocalin 2 [Source: VGNC Symbol; Acc: VGNC:30814]	3.45	2.62E-15
*IL12B*	Interleukin 12B [Source: VGNC Symbol; Acc: VGNC:30111]	3.45	1.44E-19
*S100A9*	S100 calcium-binding protein A9 [Source: VGNC Symbol; Acc: VGNC:34247]	3.42	2.30E-21
*S100A8*	S100 calcium-binding protein A8 [Source: VGNC Symbol; Acc: VGNC:34246]	3.42	2.11E-19
*RAB20*	RAB20, member RAS oncogene family [Source: NCBI gene; Acc: 615760]	3.33	3.22E-32
*HP*	Haptoglobin [Source: NCBI gene; Acc: 280692]	3.29	2.42E-15
*DEFB10*	Beta-defensin 10 [Source: NCBI gene; Acc: 100141457]	3.28	2.52E-14
*HBB*	Hemoglobin, beta [Source: NCBI gene; Acc: 280813]	−3.74	9.97E-25
*ALAS2*	5′-Aminolevulinate synthase 2 [Source: VGNC Symbol; Acc: VGNC:25804]	−3.80	3.14E-32
*HBA2*	Hemoglobin, alpha 2 [Source: NCBI gene; Acc: 512439]	−4.86	3.14E-22
*HBA1*	Hemoglobin, alpha 1 [Source: NCBI gene; Acc: 100140149]	−4.88	2.13E-22

**TABLE 2:** Summary of differentially expressed (DE) genes between bovine respiratory disease (BRD) clusters.

	**Cluster comparison^1^**		
**Item**	*B–A	*C–A	*C–B
Total transcripts	13,404	13,404	13,404
↑ Expression	1,739	3,806	581
↓ Expression	1,670	3,464	1,472
Total DEG	3,409	7,270	2,053
No significant changes	9,995	6,134	11,351

^1^Cluster with (*) annotation is upregulated compared with opposite cluster. Transcripts in B upregulated compared with A. Transcripts in C upregulated when compared with A. Transcripts in C upregulated when compared with B.

### Analysis of Bovine Respiratory Disease Clusters and Differentially Expressed Genes

**FIGURE 2:**
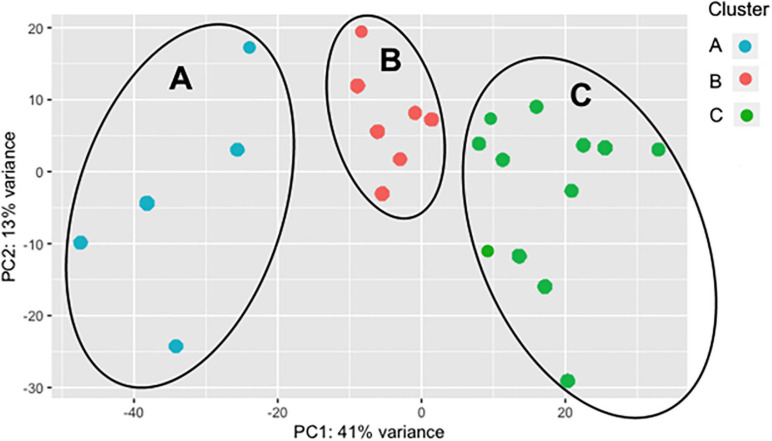
Principal component analysis (PCA) displaying clustering of bovine respiratory disease (BRD) samples. Cluster dendrogram identifying groups in the BRD population (*n* = 25) that are similar to one another based on gene expression. The BRD data is further subdivided into three distinct clusters.

As the BRD samples were more dispersed in the PCA than those from NB (Figure 1), gene expression in the 25 BRD animals was investigated further. Three distinct subsets or clusters were identified within the BRD samples (Figure 2). These clusters were not associated with serum haptoglobin level or rectal temperature at clinical examination (Supplementary Table 1).

**FIGURE 3:**
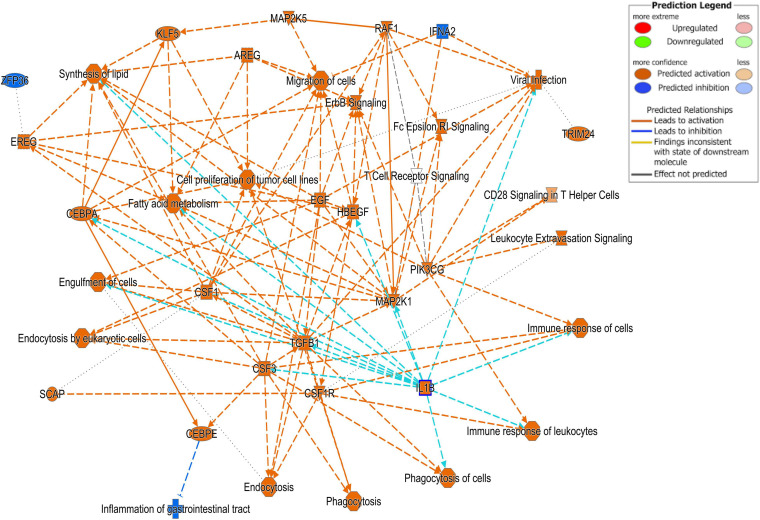
Ingenuity Pathway Analysis (IPA). Cell signaling pathways involved in the viral response pathway identified as one of the top disease pathways according to z-score in the comparison between Cluster A animals and Clusters B and C.

Differentially expressed gene values were calculated within the BRD samples (*n* = 25) and compared with one another for DEG profile, with cluster used as the coefficient to determine DEGs; log2FC > 2, *P*-adj < 0.05. A total of 13,404 DEGs were identified in these samples (Table 2). As Cluster A appeared to be the most distinct, Cluster A read counts were compared with those in Clusters B and C. With the use of logFC > 2, *P*-value < 0.05, *P*-adj < 0.01, when compared with A, 109 DEGs common to Clusters B and C were identified (34 upregulated and 74 downregulated). There were 273 DEGs unique to Cluster C and 18 to Cluster B when compared with Cluster A. The top upregulated genes unique to Cluster B included *multidrug resistance protein 4*, *duodenase-1*, and *trefoil factor 2*, while the top downregulated genes were all from the keratin family (Table 3). For Cluster C, upregulated genes included *cornifin B-like*, *solute carrier family 6*, and *serine protease 50*, while *thy-1 cell surface antigen* and *leucine-rich alpha-2-glycoproteins* were downregulated (Table 3). When compared with animals in Cluster B and C, animals in Cluster A showed increased expression of genes encoding bovine antimicrobial peptides. Specifically, *cathelicidin-2* (*CATH2*), *CATH3*, *CATH5*, and *CATH6* were upregulated in Cluster A (Table 4). These genes had high logFC values (>log2), and genes for other antimicrobial peptides such as *enteric beta defensin* (*EBD*) and *beta-defensin 4A* (*DEFB4*) were also upregulated in Cluster A, when compared with those in B and C. Genes downregulated in Cluster A when compared with Cluster B and C are shown in Table 5. Further analysis using the core analysis function in IPA shows the pathway involved in viral infection as one of the top disease pathways according to z-score in the comparison between Cluster A animals with Clusters B and C (Figure 3). The highly activated genes in this comparison include *LCN2*, *S1008A*, and *CFB*, with *bovine cathelicidin antimicrobial peptide* (*CAMP*) having the highest experimental log ratio value as identified through IPA (Table 6).

**TABLE 3:** Unique genes of interest in Clusters B and C.

**Cluster B**		**Cluster C**	
**Upregulated**	**Downregulated**	**Upregulated**	**Downregulated**
*LOC521568: Multidrug resistance associated protein 4 LOC508858: Duodenase 1 SV2C: Synaptic vesicle glycoprotein VSIG2: V-set and immunoglobulin domain containing 2 TFF2: Trefoil factor 2*	*KRT85: Keratin 85 KRT83: Keratin 83 KRT33B: Keratin, type 1 cuticular Ha3-I-like KRT33A: Keratin 33A KRT86: Keratin 86*	*LOC507527: Cornifin-B-like SLC6A15: Solute carrier family 6 ANK1: Ankyrin 1 KRT25: Keratin 25 PRSS50: Serine protease 50 BOLA-DQB: Major histocompatibility complex, class II, DQ beta*	*THY1: thy-1 cell surface antigen LOC51110: Serpin peptidase inhibitor, clade B like PLIN5: Perilipin 5 ALPL: Alkaline phosphatase, biomineralization associated LRG1: Leucine-rich alpha-2-glycoprotein*

**TABLE 4:** Top enriched bovine respiratory disease (BRD) differentially expressed (DE) genes in Cluster A compared with Clusters B and C.

**Gene name**	**Gene description**	**LogFC**	***P*-Adjust**
*CATHL2*	Cathelicidin 2 [Source: NCBI gene; Acc: 282165]	8.91	1.03E-33
*CD177*	CD177 molecule [Source: VGNC Symbol; Acc: VGNC:27006]	8.35	1.17E-34
*CATHL6*	Cathelicidin 6 [Source: NCBI gene; Acc: 317651]	7.71	2.28E-33
*CATHL3*	Cathelicidin 1 [Source: NCBI gene; Acc: 282164]	7.37	2.77E-26
*CATHL5*	Cathelicidin 5 [Source: NCBI gene; Acc: 282167]	7.04	1.48E-28
*NGP*	Neutrophilic granule protein-like [Source: NCBI gene; Acc: 788112]	5.77	1.12E-13
*LTF*	Lactotransferrin [Source: VGNC Symbol; Acc: VGNC:31077]	5.57	1.01E-30
*MS4A3*	Membrane spanning 4-domains A3 [Source: VGNC Symbol; Acc: VGNC:58392]	5.54	1.49E-16
*EBD*	Enteric beta-defensin [Source: NCBI gene; Acc: 281743]	5.42	4.90E-20
*ORM1*	Orosomucoid 1 [Source: NCBI gene; Acc: 497200]	5.10	2.85E-23
*DEFB4A*	Defensin, beta 4A [Source: NCBI gene; Acc: 286836]	5.01	4.34E-20
*PGLYRP1*	Peptidoglycan recognition protein 1 [Source: VGNC Symbol; Acc: VGNC:32791]	5.01	6.43E-29
*MMP8*	Matrix metallopeptidase 8 [Source: VGNC Symbol; Acc: VGNC:31530]	5.00	2.50E-18
*CCL14*	Chemokine (C-C motif) ligand 14 [Source: NCBI gene; Acc: 616723]	4.57	1.16E-22
*FLT4*	fms related tyrosine kinase 4 [Source: VGNC Symbol; Acc: VGNC:29044]	4.51	9.30E-13
*EFNB2*	Ephrin B2 [Source: VGNC Symbol; Acc: VGNC:28360]	4.34	2.04E-23
*IL1R2*	Interleukin 1 receptor type 2 [Source: VGNC Symbol; Acc: VGNC:30132]	4.32	8.21E-16
*MMP27*	Matrix metallopeptidase 27 [Source: VGNC Symbol; Acc: VGNC:54886]	4.25	8.38E-08
*RETN*	Resistin [Source: VGNC Symbol; Acc: VGNC:33877]	4.13	1.63E-18
*FOLR3*	Folate receptor 3 [Source: NCBI gene; Acc: 516067]	4.07	6.08E-07
*HSPG2*	Heparan sulfate proteoglycan 2 [Source: VGNC Symbol; Acc: VGNC:29988]	3.82	2.72E-22
*LCN2*	Lipocalin 2 [Source: VGNC Symbol; Acc: VGNC:30814]	3.75	6.71E-18
*MMP9*	Matrix metallopeptidase 9 [Source: VGNC Symbol; Acc: VGNC:31531]	3.65	2.20E-12
*TMEM217*	Transmembrane protein 217 [Source: VGNC Symbol; Acc: VGNC:36039]	3.53	2.21E-16
*LBP*	Lipopolysaccharide-binding protein [Source: VGNC Symbol; Acc: VGNC:56192]	3.48	4.27E-10
*RAB3IL1*	RAB3A interacting protein like 1 [Source: VGNC Symbol; Acc: VGNC:33655]	3.46	2.11E-09
*ALOX5*	Arachidonate 5-lipoxygenase [Source: VGNC Symbol; Acc: VGNC:25844]	3.34	3.28E-13
*SERPINB2*	Serpin family B member 2-like [Source: NCBI gene; Acc: 281376]	3.29	7.61E-11
*BPI*	Bactericidal permeability increasing protein [Source: NCBI gene; Acc: 280734]	3.20	6.12E-08
*CCL24*	C-C motif chemokine ligand 24 [Source: VGNC Symbol; Acc: VGNC:26950]	3.18	5.34E-10
*ITGA9*	Integrin subunit alpha 9 [Source: VGNC Symbol; Acc: VGNC:30320]	3.18	4.25E-13
*RGL1*	Ral guanine nucleotide dissociation stimulator like 1 [Source: VGNC Symbol; Acc: VGNC:33903]	3.13	1.13E-19
*EREG*	Epiregulin [Source: VGNC Symbol; Acc: VGNC:28575]	3.11	6.57E-12
*SERPINB4*	*Bos taurus* serpin peptidase inhibitor, clade B like (LOC786410), mRNA. [Source: RefSeq mRNA; Acc: NM_001206713]	3.07	1.15E-06

**TABLE 5:** Genes downregulated in Cluster A when compared with Clusters B and C.

**Gene name**	**Gene description**	**LogFC**	***P*-Adjust**
*TAC3*	Tachykinin 3 [Source: VGNC Symbol; Acc: VGNC:35556]	−5.16	7.22E-08
*LOC100139881*	Mast cell protease 2 [Source: NCBI gene; Acc: 100139881]	−3.76	4.82E-05
*FOLH1B*	Folate hydrolase 1B [Source: NCBI gene; Acc: 505865]	−3.52	5.78E-03
*LOC100847119*	Immunoglobulin lambda-1 light chain-like [Source: NCBI gene; Acc: 100847119]	−3.48	4.12E-04
*NRIP3*	Nuclear receptor interacting protein 3 [Source: VGNC Symbol; Acc: VGNC:32264]	−3.30	4.99E-04
*LARP6*	La ribonucleoprotein domain family member 6 [Source: VGNC Symbol; Acc: VGNC:30793]	−3.06	3.09E-07
*BREH1*	Retinyl ester hydrolase type 1 [Source: NCBI gene; Acc: 497207]	−2.95	1.30E-08
*GABRD*	Gamma-aminobutyric acid type A receptor delta subunit [Source: VGNC Symbol; Acc: VGNC:29198]	−2.91	2.94E-05
*SEMA3G*	Semaphorin 3G [Source: VGNC Symbol; Acc: VGNC:34432]	−2.82	1.72E-09
*KLHDC8A*	Kelch domain containing 8A [Source: VGNC Symbol; Acc: VGNC:30639]	−2.79	1.07E-08
*ADGRA1*	Adhesion G protein-coupled receptor A1 [Source: VGNC Symbol; Acc: VGNC:55933]	−2.79	2.59E-06
*PRG3*	Proteoglycan 3 [Source: NCBI gene; Acc: 617374]	−2.75	1.68E-02
*WNT5A*	Wnt family member 5A [Source: VGNC Symbol; Acc: VGNC:36960]	−2.73	2.99E-06
*GATA2*	GATA-binding protein 2 [Source: VGNC Symbol; Acc: VGNC:29266]	−2.68	1.51E-04
*GZMB*	Granzyme B (granzyme 2, cytotoxic T-lymphocyte-associated serine esterase 1) [Source: NCBI gene; Acc: 281731]	−2.65	7.07E-04
*KCNIP3*	Potassium voltage-gated channel interacting protein 3 [Source: NCBI gene; Acc: 513316]	−2.61	2.09E-12
*WC1.1*	Antigen WC1.1 [Source: NCBI gene; Acc: 786796]	−2.59	1.58E-06
*GCSAML*	Germinal center associated signaling and motility like [Source: HGNC Symbol; Acc: HGNC:29583]	−2.56	3.99E-04
*PRRS50*	Serine protease 50 [Source: NCBI gene; Acc: 518845]	−2.49	2.03E-05
*CD163L1*	CD163 molecule-like 1 [Source: NCBI gene; Acc: 338056]	−2.49	9.03E-11
*TGFB2*	Transforming growth factor beta 2 [Source: VGNC Symbol; Acc: VGNC:35802]	−2.48	7.07E-04
*CD1E*	CD1e molecule [Source: VGNC Symbol; Acc: VGNC:27008]	−2.45	1.74E-04
*CXCL12*	C-X-C motif chemokine ligand 12 [Source: VGNC Symbol; Acc: VGNC:27848]	−2.44	6.06E-12
*LY6G6C*	Lymphocyte antigen 6 family member G6C [Source: VGNC Symbol; Acc: VGNC:31090]	−2.43	8.71E-09
*KCNQ4*	Potassium voltage-gated channel subfamily Q member 4 [Source: VGNC Symbol; Acc: VGNC:30489]	−2.40	4.37E-08
*SLC6A15*	Solute carrier family 6 member 15 [Source: VGNC Symbol; Acc: VGNC:34918]	−2.39	1.71E-02
*BOLA-DQB*	Major histocompatibility complex, class II, DQ beta [Source: NCBI gene; Acc: 282495]	−2.38	7.68E-03
*CYGB*	Cytoglobin [Source: VGNC Symbol; Acc: VGNC:50268]	−2.36	7.37E-08
*ANK1*	Ankyrin 1 [Source: NCBI gene; Acc: 353108]	−2.35	5.22E-03
*RTN4RL1*	Reticulon 4 receptor like 1 [Source: VGNC Symbol; Acc: VGNC:34207]	−2.34	4.80E-08
*ENPP1*	Ectonucleotide pyrophosphatase/phosphodiesterase 1 [Source: VGNC Symbol; Acc: VGNC:28504]	−2.33	3.33E-08
*CHCHD6*	Coiled-coil-helix-coiled-coil-helix domain containing 6 [Source: VGNC Symbol; Acc: VGNC:27274]	−2.33	3.33E-08
*HRH4*	Histamine receptor H4 [Source: VGNC Symbol; Acc: VGNC:29956]	−2.33	8.85E-07

**TABLE 6:** Ingenuity Pathway Analysis (IPA) list of genes predicted to affect viral infection in Cluster A compared with B and C.

**ID**	**Genes in dataset**	**Prediction**	**Expr log ratio**	**Findings**
ENSBTAG00000024852	CAMP	Affected	8.561	Affects (1)
ENSBTAG00000001292	LTF	Affected	4.952	Affects (6)
ENSBTAG00000002635	PGLYRP1	Affected	4.617	Affects (1)
ENSBTAG00000016991	**EFNB2**^©^	**Increased**	**4.548**	**Increases** (4)
ENSBTAG00000017294	ORM1	Affected	4.39	Affects (1)
ENSBTAG00000004716	**RETN**	**Increased**	**3.478**	**Increases** (2)
ENSBTAG00000014149	**LCN2**	**Increased**	**3.092**	**Increases** (3)
ENSBTAG00000020676	MMP9	Affected	2.699	Affects (9)
ENSBTAG00000014046	BPI	Affected	2.617	Affects (1)
ENSBTAG00000020319	ALOX5	Affected	2.536	Affects (3)
ENSBTAG00000017866	**CD36**	**Increased**	**2.528**	**Increases** (7)
ENSBTAG00000006354	HP	Affected	2.511	Affects (1)
ENSBTAG00000005952	CEBPE	Affected	2.251	Affects (1)
ENSBTAG00000008059	CHRM3	Affected	2.106	Affects (3)
ENSBTAG00000048591	THBD	Affected	2.057	Affects (2)
ENSBTAG00000007169	**P2RX1**	**Increased**	**2.052**	**Increases** (2)
ENSBTAG00000039050	**P2RY2**	**Increased**	**2.051**	**Increases** (1)
ENSBTAG00000008951	ALPL	Affected	1.991	Affects (3)
ENSBTAG00000001034	IL18R1	Decreased	1.966	Decreases (2)
ENSBTAG00000012640	**S100A8**	**Increased**	**1.932**	**Increases** (4)
ENSBTAG00000021994	CACNA2D4	Affected	1.908	Affects (3)
ENSBTAG00000046152	MGAM	Affected	1.883	Affects (1)
ENSBTAG00000054057	NRG1	Affected	1.817	Affects (1)
ENSBTAG00000053072	**EFHC2**	**Increased**	**1.78**	**Increases** (1)
ENSBTAG00000014906	VCAN	Affected	1.764	Affects (1)
ENSBTAG00000040151	GCH1	Affected	1.723	Affects (1)
ENSBTAG00000038490	**CLEC4A**	**Increased**	**1.593**	**Increases** (22)
ENSBTAG00000012019	IRS2	Affected	1.544	Affects (1)
ENSBTAG00000020580	TCN1	Affected	1.538	Affects (1)
ENSBTAG00000046158	**CFB**	**Increased**	**1.519**	**Increases** (2)
ENSBTAG00000018517	**VLDLR**	**Increased**	**1.499**	**Increases** (1)
ENSBTAG00000006505	**S100A9**	**Increased**	**1.489**	**Increases** (6)
ENSBTAG00000019059	**ATG16L2**	**Increased**	**1.487**	**Increases** (2)
ENSBTAG00000012185	CLEC4E	Affected	1.474	Affects (1)
ENSBTAG00000038048	**MRC1**	**Increased**	**1.471**	**Increases** (1)
ENSBTAG00000016414	**VDR**	**Increased**	**1.468**	**Increases** (27)
ENSBTAG00000010763	**DUSP16**	**Increased**	**1.468**	**Increases** (2)
ENSBTAG00000014636	ZFHX3	Affected	1.428	Affects (1)
ENSBTAG00000006817	CBL	Decreased	1.417	Decreases (3)
ENSBTAG00000016206	MAOA	Affected	1.413	Affects (1)
ENSBTAG00000012052	**PADI4**	Increased	1.401	Increases (2)
ENSBTAG00000008592	FCGR1A	Decreased	1.382	Decreases (13)
ENSBTAG00000047338	**DCBLD1**	**Increased**	**1.327**	**Increases** (1)
ENSBTAG00000018255	ACTN1	Affected	1.318	Affects (1)
ENSBTAG00000047238	**ITGAM**	Increased	1.318	Increases (2)
ENSBTAG00000045565	NHSL2	Affected	1.316	Affects (1)
ENSBTAG00000013201	ALOX5AP	Affected	1.295	Affects (1)
ENSBTAG00000012638	**S100A12**	**Increased**	**1.264**	**Increases** (3)

*© 2000–2021 QIAGEN. All rights reserved. Bolded rows identify genes predicted to have increased activity using the IPA analysis.*

### Comparison With Related Studies

In order to determine the validity of our results, finding similarities in gene expression to related studies was also a goal of our analysis. Three studies in particular also investigated gene expression in response to cattle with BRD using the blood and bronchial lymph node transcriptome. The work done by Johnston et al. (2019) showed similarities to our work in the clear separation observed when plotting the gene expression pattern between control and infected animals, and also in the identification of genes related to acute phase protein expression (Supplementary Table 4). Additionally, Sun et al. (2020) identified enriched expression of genes belonging to heme biosynthesis, acute phase response signaling, and granzyme B signaling, which was also observed in our results (Supplementary Table 4). Finally, Scott et al. (2020), who also investigated the blood transcriptome, found similarities with the highly upregulated genes found here including *CATH2*, *LRG1*, and *CFB*, as well as decreased expression of *ALOX15* and *GZMB*.

## Discussion

Most previous studies investigating BRD have used fluids and tissues located at the main sites of infection for BRD pathogens, such as bronchial lymph nodes (Tizioto et al., 2015; Johnston et al., 2019), lung tissue (Rai et al., 2015; Chen et al., 2016; Behura et al., 2017), and lymph fluid (Gershwin et al., 2015), and have reported various immune-related genes enriched at each site of infection. In addition, these studies have collected these fluids and tissues at postmortem examination. Only a few studies (Lindholm-Perry et al., 2018; Scott et al., 2020) use RNA extracted from blood for gene expression analysis despite the relative ease of its sampling from live animals. We therefore applied a functional genomics approach to investigate changes in the whole-blood transcriptome, making two different comparisons; the first examined the difference in gene expression between all the BRD and NB animals, while the second explored the larger variation observed among the BRD animals.

As anticipated, we found that gene expression profiles in whole blood varied between animals diagnosed with BRD and those not exhibiting clinical signs of BRD. Analysis of the differential gene expression between phenotypically healthy cattle (NB) and those with BRD showed that, as with the tissues at infection sites, the major pathways activated in cattle with BRD were also associated with the host immune response.

The BRD animals also had lower expression of genes involved in hemoglobin synthesis. For example, *HBA1*, *HBA2*, *HBB*, and *ALAS2* were all downregulated in the BRD animals. These genes are involved in erythropoiesis and are regulated by iron availability (Chiabrando et al., 2014). Iron homeostasis is involved in oxygen transport, cellular respiration, and metabolic processes (Ali et al., 2017). The regulation of iron concentration in blood also plays an important role in modulating bacterial infection and contributes to the progression of lung disease (Roehrig et al., 2006; Ali et al., 2017). During bacterial infection, neutrophils maintain iron homeostasis by releasing LCN2 and lactoferrin (LTF) to sequester free iron (Ali et al., 2017) and protect the lung from oxidative stress induced by iron and HBA and HBB molecules (Tubsuwan et al., 2011). Furthermore, LCN2 decreases iron availability to limit the growth of pathogenic bacteria (Xiao et al., 2017; Pokorska et al., 2019). *Pasteurella multocida* express outer membrane protein receptors for iron-binding proteins, and the expression of these proteins increases during conditions of iron restriction (Prado et al., 2005). Animals with BRD show decreased expression of genes for hemoglobin and iron-binding proteins and regulators and an increase in genes for iron maintenance proteins (i.e., *LCN2* and *LTF*) that are released from neutrophils as a response to infection. In both comparisons of gene expression (BRD vs. NB and within the BRD animals), *LCN2* expression was increased while in the BRD vs. NB comparison, expression of genes encoding iron-binding proteins was lowered.

Bovine respiratory disease is multifactorial (Taylor et al., 2010), and etiological diagnosis of BRD is difficult if not impossible to reach in a field setting (Pardon and Buczinski, 2020). Major BRD pathogens such as *Mannheimia haemolytica*, *P. multocida*, *Haemophilus somnus*, or *Mycoplasma bovis* can be isolated from both healthy and sick animals (Angen et al., 2009; Timsit et al., 2017, 2018). Furthermore, multiple BRD pathogens (i.e., viruses and bacteria) are often detected at the same time in the same animal (Angen et al., 2009; Fulton et al., 2009), and it is impossible to determine which ones are causing lung lesions and associated clinical signs without performing a postmortem examination (Fulton and Confer, 2012). This explains why identification of the individual microbial and viral species was not performed in this study.

Although identification of the individual microbial and viral species was not performed in this study, we may be able to infer what agents were present by comparing the gene expression results with those from specific challenge studies. For example, Tizioto et al. (2015) performed single pathogen challenges with the common pathogens in the BRD complex and examined gene expression in bronchial lymph nodes of these animals (Tizioto et al., 2015). The patterns of enriched genes in the blood transcriptome in this study share similar gene characteristics with previous investigations. For example, *S100A8*, *S100A9*, and *matrix metallopeptidase 9* were highly expressed in all of the specific challenges independent of pathogen (Rai et al., 2015; Tizioto et al., 2015). An increase in expression of *S100A8* and *S100A9* is also associated with toll-like receptor 4 (TLR4) binding (Wang et al., 2016). TLR4 forms complexes that lead to recruitment of members of IL1 receptor signaling to sites of infection (Bhattacharyya et al., 2013). Interestingly we also found upregulation of *IL1R2* and *IL1RAP* in the blood of the BRD animals. Expression of *IL1* and *IL1RAP* become elevated in the host when intracellular pathogens are present (Peters et al., 2013), and both viral and bacterial pathogens can often increase the expression of this cytokine to promote a cytotoxic T cell-mediated response. We also found increased expression of *SERPINB4*, which encodes a protein located in the skin, mucous membranes, and respiratory system to prevent pathogens from crossing epithelial barriers (Geiger et al., 2015).

A second comparison analyzed the differences within the BRD samples and compared the differences between the identified clusters. Expression of several genes encoding antimicrobial peptides was increased in Cluster A compared with Clusters B and C. These included the genes such as *LTF*, and several encoding cathelicidins (*CATH2*, *CATH3*, *CATH5*, and *CATH6*). LTF functions as an antimicrobial molecule but also has immunomodulatory qualities (Drago-Serrano et al., 2017), suggesting a potential therapeutic role for this protein. Cathelicidins are defined as host defense peptides that are highly expressed in bovine granulocytes and located at mucosal surfaces in the lungs, lymphoid tissues, and intestines of the host (Baumann et al., 2017). Expression of four of the seven known bovine cathelicidin genes, *CATH2*, *3*, *5*, and *6*, was increased in the BRD animals. These peptides have been detected and isolated from sick animals and are generally not present in healthy tissues (Tomasinsig et al., 2002). Therefore, their identification as the top genes with the greatest fold-change increases in the BRD Cluster A suggests a strong host immune response in this group of affected animals. It has also been reported that *M. haemolytica* causes the induction of bovine beta-defensins, especially in animals with subacute and chronic infection (Fales-Williams et al., 2002), and we observed *enteric beta-defensin* as well as *beta-defensin 4A* among the top expressed genes in the BRD animals. It can be concluded that the expression of these defensin genes is indicative of chronic infection (Bhattacharyya et al., 2013) or simply the result of the host defense response stimulating helper T cell type 1 (TH1) and helper T cell type 2 (TH2) responses to help clear infection (Gurao et al., 2017).

The overall abundance of gamma delta T cells in ruminants is higher than in other species, and in non-ruminants, this cell subset has been associated with increasing production of TH2 cytokines (Plattner and Hostetter, 2011). Although this association has not been observed in ruminants, it has been reported that a CD163 relative, Workshop Cluster 1 (WC1), plays an important role in gamma delta T cell regulation in cattle (Herzig et al., 2010; Plattner and Hostetter, 2011), especially in young calves. This T cell subset also facilitates protective immunity following vaccinations (Davis et al., 1996; Guzman et al., 2012) and has been described to be involved in increased expression of major histocompatibility complex (MHC) class II on WC1+ cells through interaction with dendritic cells during *Mycobacterium bovis* infection (Price and Hope, 2009). When comparing Cluster A with Clusters B and C, expression of *WC1*, *WC1.1*, *WC1.3*, and *WC1-12* was significantly decreased in Cluster A. Animals in Cluster A showed lowered expression of *WC1* genes that directly promote antigen presentation and regulation of alpha beta T cells and CD4/CD8 antigens on WC1+ T cells (Ackermann et al., 2010). This suggests that the BRD animals in Cluster A were displaying lower antigen presentation and T cell regulation, suggesting that they may have been infected with a greater pathogen load that hinders the host immune response in comparison with that in the animals in Clusters B and C. Furthermore, as there was also an increase in the host antimicrobial response in Cluster A, these animals may also have had a unique pathogen subset leading to BRD than the animals in Clusters B and C.

Animals in Cluster A also exhibited a decrease in the expression of *GZMB*, which has many established roles in stimulating the cytotoxic T cell response and limiting viral replication in the host (Johnston et al., 2019). Granzyme B, in addition to leukotriene C4, IL4, and IL13, are involved in mediating allergic and asthmatic reactions in humans (Plattner and Hostetter, 2011). Basophil granulocytes are the major effector molecules in a TH2 immune response and are the source for leukotriene C4, IL4, and IL13. IL3 specifically leads to the synthesis of GZMB and contributes to the basophil granule population in the TH2 immune response (Tschopp et al., 2006), and it is one of the most potent cytokines with the longest duration of action (Tschopp et al., 2006). Therefore, the decreased expression of *GZMB* suggests that the animals in Cluster A had a lowered host immune response to infection than the animals in Clusters B and C.

## Conclusion

In conclusion, the results suggest that the blood transcriptome provides a useful resource to investigate the biology of BRD in feedlot cattle. The whole-blood transcriptome may only give a general overview of the health status, e.g., severe infection from a systemic immune response compared with that from the response reported in tissues at the site of infection. However, results from the BRD subsets (Clusters A, B, and C) do show some similarities with gene expression results using tissue and fluids isolated directly from the sites of infection, as well as other studies that also used RNA sequencing to identify BRD in tissues and blood. Analysis of the pathogens present in the sampled animals may allow this commonality to be explored further. For example, it may be that specific pathways and genes expressed in whole blood are associated with individual pathogens, which could assist in directing targeted therapeutic treatments. Such transcriptome data may also provide information on potential therapeutic targets for BRD infection. Investigation of the WC1+ cell subset and cathelicidin antimicrobial peptides could be useful in this respect. Gene expression analysis of whole blood from BRD and NB cases provides new insights for understanding host response to infection and suggests that there is significant value in using blood for BRD studies. This approach is supported by recent results obtained by Scott et al. (2020) as well as Sun et al. (2020); however, in the future, we could increase the validity of our findings by screening more animals for the genes identified in this study using qPCR. Furthermore, genes upregulated in healthy animals may also be related to protective mechanisms that reduce an individual’s susceptibility to BRD, and this warrants further investigation, as our findings put genes related to leukotriene biosynthesis and granzyme expression into this class of protective genes.

## Data Availability Statement

The RNAseq data are available at NCBI Gene Expression Omnibus (GEO) database under accession number GSE162156.

## Ethics Statement

The animal study was reviewed and approved by University of Calgary Veterinary Sciences Animal Care Committee, AC15-0109. Written informed consent was obtained from the owners for the participation of their animals in this study.

## Author Contributions

GP, ET, and KO designed the project and obtained the funding. ET designed the field trial and collected the samples. JJ prepared and analyzed the sequencing data. LG provided advice on RNAseq analysis. GP, JJ, and ET interpreted the results and drafted the manuscript. All authors contributed to the writing and revisions of the manuscript and approved the final manuscript.

## Conflict of Interest

The authors declare that the research was conducted in the absence of any commercial or financial relationships that could be construed as a potential conflict of interest.

## Footnotes

1 http://www.ingenuity.com

## Abbreviations

ADG: average daily gain
ALAS: aminolevulinic acid synthase
BoHV-1: bovine herpes virus-1
BRD: bovine respiratory disease
BRSV: bovine respiratory syncytial virus
BVDV: bovine viral diarrhea virus
CATH: cathelicidin
CFB: complement factor B
CPM: counts per million
DE: differentially expressed
DEFB: beta-defensin
DOF: days on feed
EBD: enteric beta defensin
FDR: false discovery rate
GLM: general linear model
GZM: granzyme
HB: globin
HP: haptoglobin
IL: interleukin
LCN: lipocalin
LTF: lactoferrin
MHC: major histocompatibility complex
MMP: matrix metallopeptidase
NB: non-BRD
PCA: principal component analysis
PCR: polymerase chain reaction
PI3V: bovine parainfluenza-3
RIN: RNA integrity value
SERPINB: serpin peptidase inhibitor
S100A: S100 calcium-binding protein
TLR: toll-like receptor
TMM: trimmed mean of *M*-values
TNFAIP: tumor necrosis factor alpha induced protein
WC: workshop cluster.
