# Supplementary material for: Whole-Blood Transcriptome Analysis of Feedlot Cattle With and Without Bovine Respiratory Disease
Source: Front Genet. 2021 Mar 8;12:627623. doi: 10.3389/fgene.2021.627623 (PMC7982659; doi:10.3389/fgene.2021.627623)
Supplement: Supplementary file 1 [file Data_Sheet_1.zip › Table 4.docx]

**Supplementary Table S4.** Parallel genes and functions identified in other studies that coincide with the results.

| **Article** | **Canonical Pathway** | **Genes** |
| --- | --- | --- |
| **Johnston et al. (2019)** | Acute Phase response signaling | *LRG1*🡹*, CFB* 🡹*, BOLA-DQB*🡹 |
| **Sun et al. (2020)** | **Entry-Closeout:**  Heme biosynthesis II  Leukotriene biosynthesis  TR/RXR activation  **Pulled-Closeout:**  Atherosclerosis signaling:  Acute phase response signaling  Iron homeostasis signaling  Granzyme B signaling  Differential regulation of cytokine production in intestinal epithelial cells by IL-1 | *ALAS2*  *ALOX5*  *HP*  *ALOX15, S100A8, ALOX5*  *HP, CFB, IL1R1*  *HP, PDGFRA, HBD, ALAS2*  *GZMB*  *LCN2* |
| **Scott et al. (2020)** | Inflammation regulation, innate immunity and epithelial wound healing | *ALOX15*🡻, *GZMB*🡻,  *CFB* 🡹, *LRG1*🡹 *CATH2*🡹 |

^1^ Canonical pathways either identified within the text of the referenced paper or through data obtained from supplemental material identifying differentially expressed genes.
