# Supplementary material for: Whole-Blood Transcriptome Analysis of Feedlot Cattle With and Without Bovine Respiratory Disease
Source: Front Genet. 2021 Mar 8;12:627623. doi: 10.3389/fgene.2021.627623 (PMC7982659; doi:10.3389/fgene.2021.627623)
Supplement: Supplementary file 1 [file Data_Sheet_1.zip › Table 3.docx]

**Supplementary Table S3. Summary of total reads and average reads mapped using STAR alignment for NB and BRD samples**

|  | **Treatment** | |
| --- | --- | --- |
| **Reads** | NB | BRD |
| **Total** | 724, 937, 249 | 780, 756, 072 |
| **Mean input/sample** | 31, 468, 812 | 29, 824, 901 |
| **Mean uniquely mapped/sample** | 25, 784, 647 | 24, 544, 285 |
| **Mean assigned/sample** | 24, 282, 909 | 23, 191, 001 |
